# Anatomical Positions of Superior Parathyroid Gland with regard to the Zuckerkandl Tubercle in Patients Undergoing Thyroidectomy in a Tertiary Care Centre: A Descriptive Cross-sectional Study

**DOI:** 10.31729/jnma.6012

**Published:** 2021-02-28

**Authors:** Deepak Regmi, Rachana Baidhya, Ashik Rajak, Nain Bahadur Mahato, Sangita Shrestha, Meera Bista

**Affiliations:** 1 Department of Otorhinolaryngology Head and Neck Surgery, Kathmandu Medical College Teaching Hospital, Kathmandu, Nepal

**Keywords:** *hypoparathyroidism*, *parathyroid gland*, *recurrent laryngeal nerve*, *Zuckerkandl tubercle*

## Abstract

**Introduction:**

Zuckerkandl tubercle is a prominent anatomical structure of the thyroid lobe. Identification and preservation of recurrent laryngeal nerve and parathyroid glands during thyroid surgery can be made easier through assessment of their relationship with the Zuckerkandl tubercle. This study aims to determine the anatomical relationship between Zuckerkandl tubercle and superior parathyroid in patients who underwent thyroidectomy in a tertiary care center.

**Methods:**

This descriptive cross-sectional study was conducted at a tertiary care hospital of Nepal following ethical clearance from the Institutional Review Committee (Reference no: 0106201804) among patients who underwent thyroid surgery between July 2018 to February 2020. Convenience sampling was used for collecting data and was entered in Statistical Package for the Social Sciences version 20. Point estimate at 95% confidence interval was calculated along with frequency and proportion for binary data.

**Results:**

Out of 59 cases, 27 (96.4%) of superior parathyroid on the left were at the 1-2 o'clock position, and 28 (90.3%) of superior parathyroid on the right were at 10-11 o'clock position. On the left side, the superior parathyroid was adhered to Zuckerkandl tubercle in 10 (35.7%), was within 5 mm in 16 (57.1%), and was >5 mm away from Zuckerkandl tubercle in 2 (7.14%). On the right side, the superior parathyroid was adhered to Zuckerkandl tubercle in 12 (38.7%), was within 5 mm in 13 (41.93%), and was >5 mm away from Zuckerkandl tubercle in 6 (19.3%).

**Conclusions:**

Zuckerkandl tubercle has a consistent relationship with the superior parathyroid and can be used as an important landmark for identifying superior parathyroid during thyroid surgery.

## INTRODUCTION

A thorough understanding of the anatomy of the thyroid, parathyroid and associated neurovascular structures has enhanced the efficacy and safety of thyroid surgeries.^1,2^ Parathyroid mobilization during thyroid surgery can cause transient or permanent hypoparathyroidism.^3,4^ Failure to recognize and preserve this gland may lead to increased risk of inadvertent excision^5^ or parathyroid gland injury, with an incidence ranging from 17%6 to 19.8%.^7^

Zuckerkandl tubercle (ZT) is the most prominent area in the posterolateral aspect of the thyroid lobes^8^. On identification, its consistent relation with recurrent laryngeal nerve (RLN) and superior parathyroid gland (SP)^9^ can prove helpful for safe dissection.^10^ While many studies have described the association between ZT and RLN, there are less studies reporting the anatomical relation between ZT and SP.^8^

This study aims to determine the anatomical relationship between Zuckerkandl tubercle and superior parathyroid in patients who underwent thyroidectomy in a tertiary care center.

## METHODS

This is a descriptive cross-sectional study carried out in patients undergoing thyroid surgery in Kathmandu Medical College and Teaching Hospital, Kathmandu, Nepal. The study was conducted from July 2018 to February 2020. Ethical approval was taken from the Institutional Review Committee of Kathmandu Medical College and Teaching Hospital (Reference no: 0106201804). All the patients who underwent thyroidectomy of any type in which ZT was identified were included in the study. The patient who had undergone previous radiation and thyroid cancer with gross extrathyroidal spread where ZT was difficult to locate or else involved by the tumor were excluded from the study. Informed written consent was taken from the participants. A convenience sampling technique was used. The sample size was calculated by using the formula,


$$\begin{array}{ll} \text{n} & ={\text{Z}}^{\text{2}}\times \text{p}\times \text{q}/{\text{e}}^{\text{2}}\\ & ={\left(1.96\right)}^{2}\times 0.96\times 0.04/0.05^{\text{2}}\\ & =59 \end{array}$$

Where,

n = sample size,
Z = 1.96 at 95% confidence level,
p = prevalence (0.96)^8^,
q = 1-p,
e = margin of error (5%).

A detailed history was taken and a clinical examination of the thyroid swelling was done. The pre-operative assessment was done with the ultrasonography (USG) of the neck, thyroid function test, fine needle aspiration cytology (FNAC) and fiber-optic Naso-pharyngolaryngoscopy (NPL). Each side of the neck was taken as a separate entity. Under the aseptic condition, all the operations were performed according to standard surgical technique.^11^

ZT, if present, was identified and graded according to Pelizzo's grading system into four types as grade 0, grade I, grade II and grade III.^12^ Grade 0 ZT is unidentified and was excluded from our study, grade I (ZT width ≤ 5 mm), grade II (ZT width 6-10 mm) and grade III (ZT width >10 mm) were only included in the study. The width of the ZT was defined as the longest length between the upper and lower margins of the ZT. Localization and preservation of parathyroid glands were done. Relation of the SP with ZT was assessed. The sterile measuring tape was used for measuring the distance between ZT and SP. The distance was measured from the lateral most part of the ZT to the point of nearest distance to the SP. The relation between them and the distance measured was recorded. The data were entered into Microsoft Excel and analyzed in the Statistical Package for the Social Sciences (SPSS) version 20.

## RESULTS

ZT was identified and graded according to Pelizzo's grading.^12^ The size of the ZT was taken as the greatest width of ZT. On the right side, 8 (20.5%) of the ZTs were grade 0, 5 (12.8 %) were grade I, 20 (51.28 %) were grade II and 6 (15.38%) were grade III. On the left side, 7 (20%) of the ZTs were grade 0, 5 (14.28%) were grade I, 18 (51.4%) were grade II and 5 (14.28%) were grade III.

**Table 1:** Site wise grade of ZT.

Grade	n (%)			
	0 n (%)	I n (%)	II n (%)	III n (%)
Right (n=39)		8 (20.5) 5 (12.8)	20 (51.28)	6 (15.38)
Left (n= 35)	7 (20) 5 (14.28)	18 (51.4)	5 (14.28)

SP was located in relation to ZT on the right and left side in a clock-face. Among the 59 cases, 7 (25 %) of SPs were in the 1 o'clock position, 20 (71.4%) were in the 2 o'clock position, and 1 (3.5%) was in the 3 o'clock position on the left side. On the right side, 3 (9%) of SPs were in the 9 o'clock position, 18 (58.06%) were in the 10 o'clock position, 10 (32.25%) were in the 11 o'clock position, and none were detected on the anterior surface of the ZT.

**Table 2:** Distance between the ZT and the SP.

Distance between the ZT and the SP n (%)			
Site	0 mm n (%)	< 5mm n (%)	≥ 5mm n (%)
Left (N=28)	10 (35.7)	16 (57.1)	2 (7.14)
Right (N=31)	12 (38.7)	13 (41.93)	6 (19.3)

In this study, the distance between ZT and the SP was measured. On the left side, the SP was adhered to the ZT in 10 (35.7%), was within 5 mm of the ZT in 16 (57.1%) and was >5 mm away from ZT in 2 (7.14%). On the right side, the SP was adhered to the ZT in 12 (38.7%), was within 5 mm of the ZT in 13 (41.93%) and was >5 mm away from ZT in 6 (19.3%).

The relationship between the size of the ZT and its distance from SP was analyzed and it showed an inverse relationship between the width of ZT and its distance from the SP.

**Table 3:** Relationship between the ZT width and the SP.

		ZT width				
	≤ 5mm		6-10mm		>10mm	
Distance	Right	Left	Right	Left	Right	Left
from SP	n (%)	n (%)	n (%)	n (%)	n (%)	n (%)
0 mm	0 (0)	0 (0)	6 (30)	5 (27.7)	6 (100)	5 (100)
<5mm	3 (60)	4 (80)	10 (50)	12 (66.6)	0 (0)	0 (0)
≥ 5mm	2 (40)	1 (20)	4 (20)	1 (5.5)	0 (0)	0 (0)

## DISCUSSION

One of the major concerns during thyroid surgery is identifying and preserving the parathyroid glands as their injury results in lower quality of life.^8^ Therefore, in-depth knowledge of anatomical relations, the variation of the thyroid and parathyroid glands with their vascular supply and relationship with laryngeal nerves is the cornerstone for performing safe thyroid and parathyroid surgery.^8^

Embryologically, the thyroid gland originates as a fusion of the median and the lateral thyroid anlages.^8,9^ The embryology of the median anlage is better understood. It begins as a ventral diverticulum of the pharyngeal endoderm, which later forms a bilobed structure and descends anteriorly to the trachea. By seven weeks, it reaches its final position.^13^ Two lateral anlages (ultimobranchial bodies) develop as an evagination of the ventral portion of the endoderm of the fourth branchial pouch and the vestigial fifth pouch.^14^ As the embryo grows and reaches the length of 10 mm, there is a parathyroid primordium adjacent to each lateral thyroid component. Further longitudinal growth of median and lateral thyroid components leads to detachment of pedicles of lateral primordia from the pharynx, which is then replaced by mesenchyme. This residual posterolateral projection towards the pharynx from the lateral thyroid component, when present is the ZT named after Emil Zuckerkandl in 1902, is an important landmark in thyroid and parathyroid surgery.^9^

The SPs develop from the fourth pharyngeal pouch,^15^ and after being separated from the pharyngeal wall, they connect to the posterior surface of the caudally migrating thyroid.^2^ It has much shorter embryological descent than the inferior parathyroid gland (IP), leading to smaller variability in location than IPs.^2,16^

Yun et al.^8^ reported a constant anatomical relationship between ZT and SP and emphasized the ZT as an important landmark for the preservation of SP. Generally, SPs are located at the posterior aspect of the thyroid lobe in a 2 cm diameter area centered 1 cm above the crossing of the inferior thyroid artery (ITA) and RLN,^17^ and cranial to ZT.^3^ In our study, we found that 96.4% of SPs on the left and 90.3% of SPs on the right are cranial to ZT (mostly in 1 and 2 o'clock positions on the left and 10 and 11 o'clock position on the right), indicating a constant relationship between ZT and SP. Similar findings were also reported in a previous study by Yun et al.^8^ which showed that 95.6% of SPs are located between 1-2 o'clock and 10-11 o'clock position to ZT and Gauger et al.^9^ which showed that SPs are consistently found in a cranial position in relation to ZT.

The present study reported that wider ZTs are nearer to SPs (adhered or at <5 mm distance) and narrower ZTs are farther to SPs (at > 5 mm distance). Therefore, once the ZTs are identified, a search of the SPs should not be missed in the cranial portion of ZTs (especially at 1 and 2 o'clock position on left and 10 and 11 o'clock position on the right) and whenever the identified ZT is greater than 5 mm in width, exploration and preservation of the SPs should be thought from the time of dissection of the ZT capsule. By doing this, we can reduce the inadvertent excision of the SPs and hence also significantly decrease the incidence of permanent hypoparathyroidism and related complications.^4^

Certain limitations were present in our study. It was carried out in a single institute with small sample size. Therefore, more similar studies need to be done with a larger sample size before generalizing it to the general population.

## CONCLUSIONS

The study finds a consistent anatomical relationship between ZT and SP, when ZT is identified. In spite of the fact that ZT is identifiable in most thyroid surgeries and has an important relationship with SP and RLN, the knowledge has not yet been circulated enough. Through this study, ZT can be considered an important anatomical landmark for preserving the SP during thyroid surgery.
